# Use of Distal Tibial Cortical Bone Thickness and FRAX Score for Further Treatment Planning in Patients with Trimalleolar Ankle Fractures

**DOI:** 10.3390/jcm12113666

**Published:** 2023-05-25

**Authors:** Patrick Pflüger, Felix N. Harder, Karoline Müller, Lukas Willinger, Peter Biberthaler, Moritz Crönlein

**Affiliations:** 1 Department of Trauma Surgery, Klinikum Rechts der Isar, Technical University of Munich, Ismaninger Str. 22, 81675 Munich, Germany; 2 Institute of Diagnostic and Interventional Radiology, School of Medicine, Technical University of Munich, 81675 Munich, Germany; 3 Department of Orthopaedic Sports Medicine, Klinikum Rechts der Isar, Technical University Munich, 81675 Munich, Germany

**Keywords:** ankle fracture, fragility fracture, geriatric trauma, osteoporosis

## Abstract

Trimalleolar ankle fractures show a bimodal age distribution, affecting younger men and older women. Postmenopausal women often exhibit low bone mineral density, which contributes to a higher prevalence of osteoporotic-related fractures. The primary goal of this study was to analyse the association of patient characteristics with the cortical bone thickness of the distal tibia (CBTT) in trimalleolar ankle fractures. Methods: A total of 193 patients with a trimalleolar ankle fracture treated between 2011 and 2020 were included. Patient registries were reviewed regarding demographics, mechanism, and type of injury. The CBTT was assessed in radiographs and CT images. The FRAX score was calculated to estimate the probability for an osteoporotic fracture. A multivariable regression model was calculated to identify independent variables affecting the cortical bone thickness of the distal tibia. Results: Patients older than 55 years were 4.22 (95% CI: 2.12; 8.38) times more likely to be female. In the multivariable regression analysis, female sex (β −0.508, 95% CI: −0.739; −0.278, *p* < 0.001) and a higher age (β −0.009, 95% CI: −0.149; −0.003, *p* = 0.002) were independent variables associated with a lower CBTT. Patients with a CBTT < 3.5 mm had a higher 10-year probability for a major osteoporotic fracture (12% vs. 7.75%; *p* = 0.001). Conclusions: The assessment of the peripheral bone quality in routine computed tomography demonstrated that higher age and female sex are significantly associated with reduced cortical bone thickness of the distal tibia. Patients with a lower CBTT showed a higher probability for a subsequent osteoporotic fracture. In female patients with reduced distal tibial bone quality and associated risk factors, an osteoporosis assessment should be evaluated.

## 1. Introduction

The incidence of trimalleolar ankle fractures increased over the last decade, reaching 40 per 100,000 people per year [1]. Trimalleolar fractures show a bimodal age distribution most frequently affecting men under the age of 40 years and women above the age of 60 years [1,2,3,4]. The most common cause of injury is low-energy trauma, and falls from a standing position account for over two-thirds of all cases, especially in older women [4,5,6]. To date, trimalleolar ankle fractures are not considered as typical fragility fractures, despite the use of this term in elderly patients with unstable ankle fractures [1,7,8].

Fragility fractures are the result of inadequate trauma in patients with osteoporotic bone [9]. Almost 1 in 4 women aged 50 years and older are affected by osteoporosis, and an osteoporotic fracture can impair the quality of life and increase mortality [10]. Patterson et al. demonstrated that cortical bone thickness of the distal part of the tibia predicts bone mineral density [11]. Studies analysing the correlation between computed tomography (CT) and dual X-ray absorptiometry (DXA) showed that CT can serve as a screening tool for osteoporosis, but data for trimalleolar ankle fractures are missing [12,13,14].

A trimalleolar ankle fracture is considered unstable and treatment is generally performed surgically [15]. The posterior malleolus gained enormous importance in the last decade since several studies found an association between the presence of posterior tibia involvement and an inferior outcome [16]. Nowadays, treatment algorithms focus on the type of posterior malleolar fracture [17]. Considering the peak occurrence of trimalleolar fractures around the age of 60, generally the goal of the treatment is to restore the anatomy and congruence of the ankle joint allowing the best possible function to be regained [6,15]. However, with higher age, functional demand can differ significantly and comorbidities may lead to adverse outcome [18]. Osteopenia can contribute to a loss of fixation, leading to revision surgery and a mal-/non-union [7,19]. By initiating osteoporosis therapy, the occurrence of other fractures could be prevented and the overall occurrence of complications reduced [20].

The primary goal of this study was to analyse the patient characteristics, mechanism of injury and the cortical bone thickness of the distal tibia in trimalleolar ankle fractures.

## 2. Materials and Methods

A retrospective analysis of patients treated for a trimalleolar ankle fracture between 2011 and 2020 at the level I major trauma centre of the University Hospital rechts der Isar (Munich, Germany) was performed. Patients > 18 years with preoperative radiographs and CT images were included.

This retrospective chart review study involving human participants was in accordance with the ethical standards of the institutional and national research committee and with the 1964 Helsinki Declaration and its later amendments or comparable ethical standards. The Human Investigation Committee (IRB) of the local university approved this study (No: 600/21 S).

### 2.1. General Data

Patients’ medical records were reviewed, and the following data were obtained: age, sex, body mass index (BMI), nicotine abuse/alcohol abuse (cigarettes per day, >1 L beers per day), diabetes mellitus, malignancies (previous or active), cardiovascular disease (hypertension, coronary heart disease, heart failure, arrhythmias), rheumatoid arthritis, chronic obstructive pulmonary disease (COPD)/asthma, osteopenia/osteoporosis, preinjury mobility, and need of walking aids. 

The type of injury and concomitant lesions were retrieved from patient records and distinguished between isolated injury, polytrauma, accompanying foot injuries, ankle dislocation, open fracture, traumatic nerve injury, and traumatic vascular injury. Trauma mechanism was classified as follows: ankle distortion while walking, ankle distortion during sport, bicycle accident, motorbike accident, fall >3 m, road accident as a pedestrian, road traffic accident. To determine the burden of the injury for the hospital, the time to definite surgery (TTDS), length of hospital stay (LOS), and necessity of intensive care unit treatment were analysed. Causes of re-operation were investigated in patients with a follow-up of one year after definitive surgery.

The fracture risk assessment tool (FRAX) was used to estimate the 10-year probability of fracture [21]. Only patients between 40 and 90 years and low energy trauma were included in the analysis. The FRAX score for a major osteoporotic fracture was calculated twice to compare the impact of defining the trimalleolar ankle fracture as a previous fracture (“fracture arising from trauma which, in a healthy individual, would not have resulted in a fracture”). A history of hip fracture in the patient’s mother or father could not be assessed and, therefore, “No” was chosen for all patients. The calculation without BMD is reliable, but the fracture risk might be overestimated [22].

### 2.2. Radiographic Analysis

**Figure 1:**
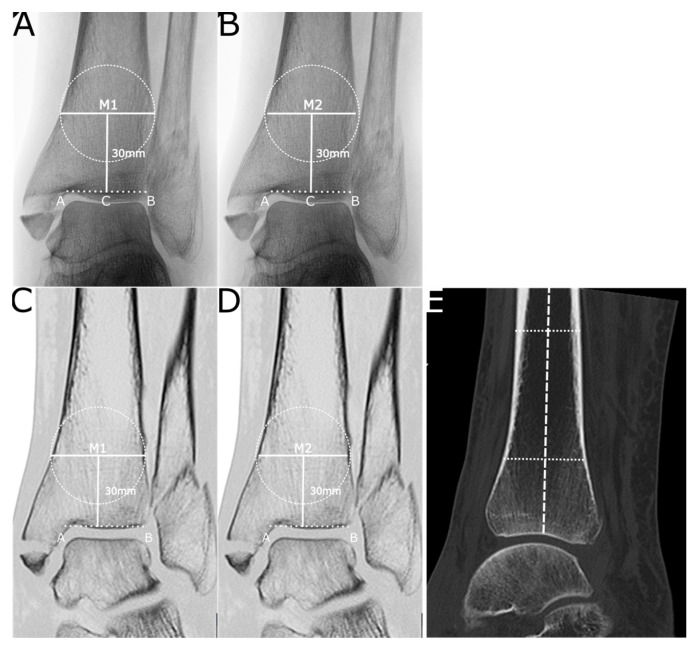
(**A**) AP radiograph showing the method of measurement of the cortical bone thickness at 30 mm proximal to the tibial joint line according to Patterson et al. [11]. The cortical bone thickness was calculated as the difference of M2 and M1 at 30 mm (R1) and 50 mm (R2). (**B**) The same method was applied in coronal CT images of the same ankle joint at 30 mm and 50 mm (**C**,**D**). In the sagittal view (**E**), the middle of the tibia was determined, and the corresponding coronal view used to measure the cortical bone thickness. The layer thickness of the CT images was at least 3 mm.

Preoperative images were analysed to determine the cortical bone thickness and trabecular density of the distal tibia. Blinded plain radiographs and CT images were assessed to determine the cortical bone thickness of the distal tibia (CBTT). The method described by Patterson et al. was used to measure the cortical bone thickness at 30 mm (R1) and 50 mm (R2) proximal to the distal tibial joint line on anteroposterior (AP) radiographs (Figure 1) [11]. For further analysis, the mean of R1 and R2 was calculated to receive the CBTT_R_. The same measurements were performed in coronal CT images at the centre of the sagittal tibial shaft axis at 30 mm and 50 mm above the ankle joint line (Figure 1) to calculate the CBTT_C_. The agreement between radiographic and CT measurements was calculated.

**Figure 2:**
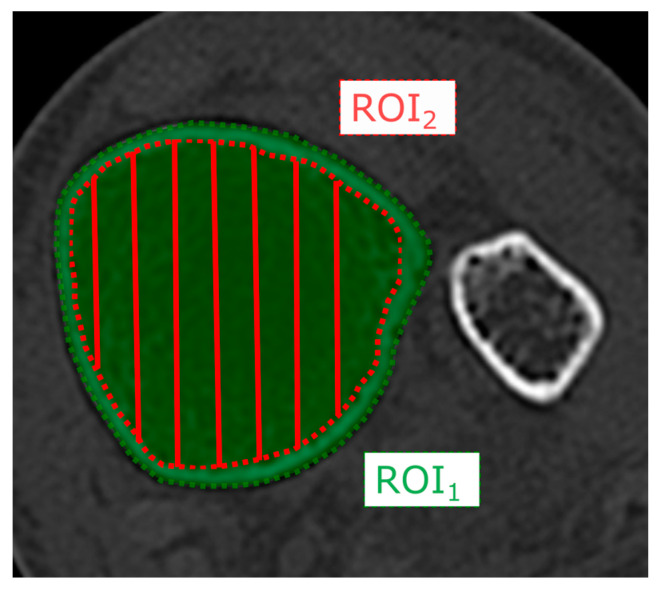
Axial CT image illustrating the fitted region of interest (ROI) to determine the tibial grey scale value of the distal tibia (ROI_1_) and the cancellous bone (ROI_2_) in Hounsfield units (HUs).

Grey scale values measured in Hounsfield Units (HUs) were determined in axial CT scans of the distal tibia (ROI_1_) and the cancellous bone (ROI_2_). The regions of interest (ROIs) were manually defined by the investigator 30 mm above ankle joint line (Figure 2).

### 2.3. Statistics

Data are presented as a mean ± standard deviation (SD) or median and interquartile range (IQR). RStudio (R version 3.6.2 (12 December 2019), PBC, Boston, MA, USA) was used for data processing and a *p*-value < 0.05 was considered statistically significant. 

A quantile–quantile (Q–Q) plot was used to assess data normality.

A multivariable regression model was calculated using cortical bone thickness of the distal tibia as a dependent variable. Backward stepwise selection was used to select the important variables. The variables tested included age, sex, BMI, nicotine/alcohol abuse, diabetes mellitus, malignancies, cardiovascular disease, chronic obstructive pulmonary disease (COPD), and asthma. The results are reported as odds ratios (ORs) with their 95% confidence intervals (CIs). Adjusted R^2^ was calculated as a measure of the goodness of fit of the models.

As appropriate, the non-parametric Mann–Whitney U test or the parametric t-test was used to assess significant differences between two groups and the Kruskal–Wallis test or analysis of variance (ANOVA) in the case of more than two groups. Post hoc analysis was calculated with Bonferroni correction. The correlation between the different CT measurements was assessed with the Pearson correlation coefficient and agreement between radiographs and CT with the Bland–Altman plot. To calculate the strength and association of two categorical variables, the OR and 95% CI was calculated. The chi-squared test was used to assess significant differences between the categorical variables.

## 3. Results

### 3.1. General Data

A total of 193 patients with a trimalleolar ankle fracture were included in this study. The baseline data of the patients are illustrated in Table 1. Of them, 72% were women, and patients older than 55 years were 4.22 (95% CI: 2.12; 8.38) times more likely to be female. Women had a mean age of 59.5 ± 18.2 years and men of 48.5 ± 15.6 years (*p* < 0.001). A history of cardiovascular disease was present in 38%, but previously diagnosed osteopenia or osteoporosis was rare. Five women had known osteoporosis and three women had an osteopenia. Patients with known osteopenia were treated with basic treatment (vitamin D and calcium supplementation), and patients with osteoporosis received specific anti-osteoporotic medication. The majority of patients were able to ambulate independently without limitation or the need of a walking aid (Table 1).

The most frequent cause of injury was an ankle joint distortion during walking, whereas high-energy trauma tended to be very rare (Table 2). Patients sustaining a low-energy ankle distortion were significantly older (*p* < 0.001) and 2.35 (95% CI: 1.15; 4.79) more likely to be female. An ankle dislocation occurred in almost ¾ of the cases, and 116 patients (60%) required acute temporary external fixation. Most of the patients sustained an isolated trimalleolar ankle fracture. Open fractures, traumatic nerve or vascular injuries were rare (Table 2). Open injuries were seen after low-energy ankle distortions (*n* = 5), bicycle accidents (*n* = 2), a motorbike accident (*n* = 1), and a road traffic accident (*n* = 1). All patients with an open trimalleolar ankle fracture following a low-energy ankle twist were older than 60 years of age.

In total, 179 patients were available for the in-hospital data analysis. Only two of these patients needed intensive care unit treatment postoperatively. The mean TTDS was 8.2 ± 5.6 days, and the LOS was 13.7 ± 7.8 days. LOS was significantly increased in patients over 45 years of age (*p* = 0.01) and in patients with cardiovascular disease (*p* < 0.001). Patients with a dislocation of the ankle had a significantly longer LOS (*p* = 0.005) and were 10.04 (95% CI: 4.58; 22.03) more likely to receive temporary external fixation. As a result of temporary external fixation, the TTDS was significantly increased (*p* = 0.008).

The most common cause for secondary surgery after definitive fracture fixation was syndesmotic screw removal (*n* = 44), followed by hardware removal after fracture consolidation (*n* = 38). Revision surgery was most commonly performed due to implant malposition (*n* = 5) and only once as a result of loss of reduction (Table 3).

One hundred and thirty patients met inclusion criteria and were available for calculating the FRAX score. When a trimalleolar ankle fracture was considered a “previous fracture”, the 10-year probability of a major osteoporotic fracture was significantly higher (FRAX_1_ = 9.45% (11.1), FRAX_2_ = 4.6% (6.65); *p* < 0.0001). On the basis of the FRAX_1_ calculation, 62 patients (48%) had a score ≥ 10%. 

### 3.2. Radiographic Analysis

**Figure 3:**
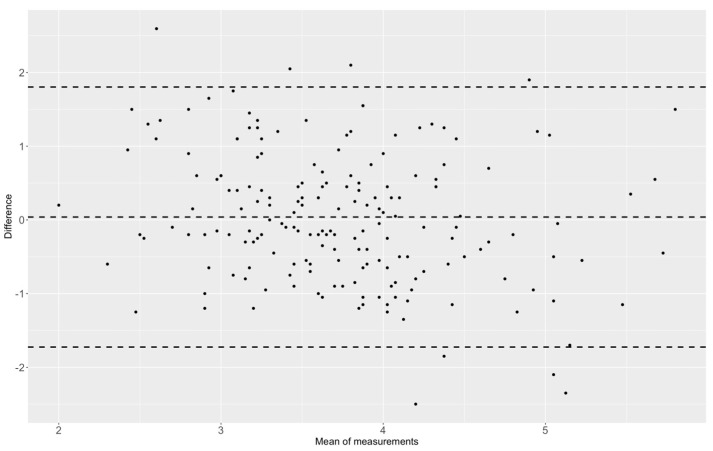
Bland–Altman-Plot showing the bias of the CBTT_C_ in comparison to CBTT_R_ values with 0.039 (lower limit: −1.73; upper limit: 1.80).

**Figure 4:**
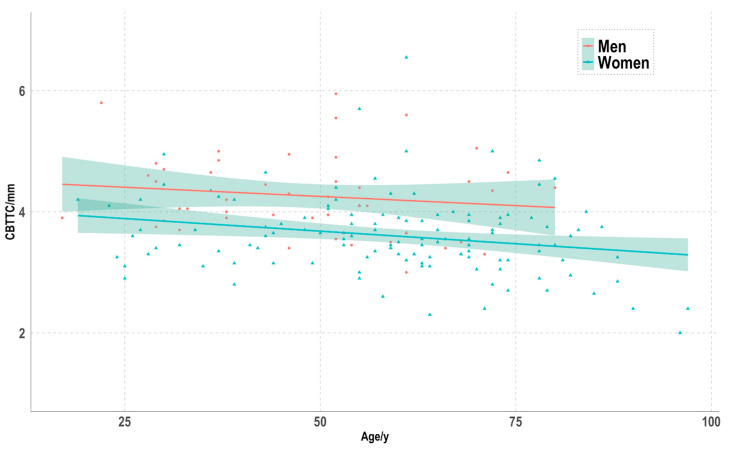
Scatterplot of the CBTTC for men and women. Regression lines of the CBTTC depending on the age, with 95% CIs.

The cortical bone thickness of the distal tibia (CBTT_R_) in standard AP radiographs was 3.6 ± 1.1 mm and CT images (CBTT_C_) were 3.8 ± 0.8 mm (*p* = 0.046). The Bland–Altman plot showed that the bias of the CBTT_C_ in comparison to CBTT_R_ values was 0.039 (lower limit: −1.73; upper limit: 1.80) (Figure 3). Due to the scattered distribution of the CBTT_R_ values in comparison to the CBTT_C_ measurements, the CBTT_C_ values were used as the endpoint in the multivariable regression model. In the multivariable regression analysis, female sex (β −0.508, 95% CI: −0.739; −0.278, *p* < 0.001) and a higher age (β −0.009, 95% CI: −0.149; −0.003, *p* = 0.002) were independent variables reducing the CBTT_C_ (Figure 4). The predictive value of the model was 0.27 (Table 4).

If setting a threshold value of 3.5 mm for the mean CBTT_C_ (Patterson et al. [11]), women were 7.17 (95% CI: 2.81; 21.84) more likely to have a lower cortical bone thickness. Regarding age (> or <45 years; OR 1.80, 95% CI: 0.87; 3.87) and mechanism of injury (=low energy; OR 1.51, 95% CI: 0.7; 3.42) the threshold value for the CBTT_C_ showed no significant differences. Patients with a CBTT_C_ < 3.5 mm had a higher 10-year probability for a major osteoporotic fracture (FRAX_1C_ = 12% (12.9), FRAX_1CD_ = 7.75% (6.9); *p* = 0.001)

The grey scale value of the distal tibia (ROI_1_) was 393.2 ± 97.8 and mean value of the cancellous bone (ROI_2_) was 180 ± 66.4. ROI_1_, and ROI_2_ showed a moderate positive correlation (0.67, 95% CI: −0.57; 0.74, *p* < 0.001). 

Multivariable regression analysis showed that age is an independent factor (β −2.325, 95% CI: −3.083; −1.565, *p* < 0.001) affecting the tibial grey value (ROI_1_), and female sex (β −29.577, 95% CI: −51.957; −7.197, *p* = 0.01) as well as age (β −29.577, 95% CI: −51.957; −7.197, *p* = 0.01) are independent variables affecting the grey value of the cancellous bone (ROI_2_). R^2^ for the ROI_1_ regression model was 0.22 and for the ROI_2_ model 0.12 (Supplements: Tables S1 and S2).

## 4. Discussion

The incidence of trimalleolar ankle fractures is rising, and, especially, elderly women are affected following low-energy trauma. Analysis of the bone quality of the distal part of the tibia in routine preoperative computed tomography demonstrated that higher age and female sex are associated with reduced cortical bone thickness of the distal tibia. If considering these ankle fractures as the first manifestation of bone fragility, the 10-year risk for a major osteoporotic fracture is significantly increased. The presence of corresponding risk factors should raise awareness for an osteoporosis assessment and adaption of treatment guidelines to prevent future osteoporotic fractures.

The demographics of the analysed patient cohort showed a higher prevalence in women and that they were at a higher risk with increasing age. This is in line with epidemiological studies showing a bimodal age distribution for trimalleolar ankle fractures with peak incidences in younger men and older women [1,2,3,4]. Because of the age and sex distribution, Court-Brown and Caesar proposed in a review that trimalleolar fractures should be regarded predominantly as osteoporotic fractures [23]. In the present cohort, only 4% of the patients received treatment for diagnosed osteoporosis prior to the injury. The high prevalence of osteoporosis of up to 25% in postmenopausal women makes a large number of undiagnosed cases likely [10]. Park et al. investigated 48 patients older than 65 years with ankle fractures and reported an osteoporosis rate of 33% in female patients diagnosed through DXA [24]. Despite the fact that the mean age of the patients was over 55 years and over half of them suffered from a comorbidity, almost 90% had no mobility restrictions before the fracture. Data on preinjury mobility in trimalleolar ankle fractures are inconclusive, but studies showed that bi-/trimalleolar ankle fractures lead to changes in gait pattern and cause lasting functional limitation [25,26,27].

The most frequent cause of injury was an ankle joint distortion during walking. These patients were, in particular, older women. Thur et al. and Elsoe et al. found in their studies that low-energy trauma is the most common cause for trimalleolar ankle fractures, especially in women over 60 years who accounted for over 2/3 of all cases [1,4]. Due to low-energy trauma, the majority of the included patients had an isolated trimalleolar ankle fracture, and concomitant injuries were rare. Nevertheless, 5% suffered an open trimalleolar ankle fracture, and the most frequent mechanism of trauma was an ankle distortion in patients above the age of 60 years. Ovaska et al. analysed demographics and complications of open ankle fractures and found comparable results regarding incidence, age, and mechanism of trauma [28]. 

The mean LOS was 14 days and significantly Increased in older patients with a cardiovascular disease and a dislocation of the ankle. Thur et al. performed an epidemiological study of adult ankle fractures and found that bi-/trimalleolar fractures had the longest LOS of all ankle fractures with an average of 10 days [4]. Other studies also found a correlation between higher age, cardiac disease, and the presence of a bi-/trimalleolar fracture with a longer LOS [29,30]. 

The multivariable regression model showed that age and sex significantly influence the distal tibial cortical bone thickness in patients with trimalleolar ankle fractures. Furthermore, the analysis demonstrated that radiographic determination of the cortical bone thickness had a large variance and low correlation with CT. Patterson et al. performed an analysis of 167 patients and concluded that the cortical bone thickness (CBT) of the distal tibia correlates with DXA findings [11]. The CBT was determined in standard ankle radiographs, and patients with a previous trauma were excluded [11]. A CBT of 3.5 mm was set as the threshold value for predicting osteoporosis, with a high sensitivity and low specificity [11]. Comparing the results to the presented data, the significance of the threshold value is rather questionable. This might be due to the problems with solely determining the CBT in radiographs and that, in DXA, bone quality is assessed in cortical as well as cancellous bone. Despite this fact, patients with a CBT of <3.5 mm showed a higher 10-year probability for a major osteoporotic fracture. Studies analysing the correlation between CT and DXA showed that computed tomography can serve as a screening tool for osteoporosis [12,13,14]. Lee KM et al. investigated 194 patients with ankle fractures and determined the mean bone attenuations of different parts of the ankle in CT examinations [13]. They showed that higher age and female sex significantly contributed to a lower bone attenuation [13]. In another study, Lee S-Y et al. found a significant correlation between bone attenuation of the distal tibia on CT and DXA, concluding that peripheral bone attenuation on lower extremity CT adequately reflects central bone mineral density (BMD) [14]. The mean age of the patients was 73 years, and only 58 ankle fractures were included [13]. In both studies, the bone attenuation of the distal tibia was evaluated by placing a circular ROI in the central part of the coronal CT image [13,14]. Therefore, the absolute values of the presented study and those of the studies of Lee KM and Lee S-Y should not be compared. Biver et al. further support the findings of the study, showing that ankle fractures in postmenopausal women are associated with low areal bone mineral density and bone microstructure alterations [12]. The authors concluded that ankle fractures are another manifestation of bone fragility [12]. This is very important and is a fact that needs to be kept in mind, since osteoporotic fractures can impair the quality of life and increase mortality [10]. In a multinational prospective study, Samelson et al. highlighted the potential benefits of the assessment of the peripheral bone quality by improving identification of people at the highest risk of fracture [20]. If considering a trimalleolar ankle fracture as the first manifestation of bone fragility, the 10-year risk for a major osteoporotic fracture significantly increases. Almost half of the patients showed a FRAX score of >10%, reaching a threshold where at least a further osteoporotic assessment should be initiated [21].

In trimalleolar ankle fractures, CT examination became a standard to assess the configuration of the posterior malleolus [15]. Therefore, the gathered information may not only be used for surgical management but may also help to close the treatment gap of osteoporosis.

Limitations of this study are its retrospective design with its associated bias. Long-term clinical and radiological results were not part of our study, and therefore no assumption can be made about the prognostic value of the cortical bone thickness of the distal tibia for the functional outcome. Due to the fact that only trimalleolar ankle fractures were included, the results cannot be transferred to other ankle fractures. No DXA scans were performed routinely as the current diagnostic gold standard for verifying osteoporosis. The FRAX score was calculated without the BMD and might, therefore, be overestimated [22]. Due to the fact that patients’ medical records were reviewed, a history of hip fracture in the patient’s mother or father could not be assessed for calculating the FRAX score.

## 5. Conclusions

Trimalleolar ankle fractures typically affect older women following low-energy ankle trauma. The assessment of the peripheral bone quality in routine computed tomography demonstrated that higher age and female sex are significantly associated with reduced bone quality of the distal tibia. If considering a trimalleolar ankle fracture as the first manifestation of bone fragility, the 10-year risk for a major osteoporotic fracture significantly increases. Furthermore, patients with a lower CBTT showed a higher probability for a subsequent osteoporotic fracture. Therefore, a trimalleolar ankle fracture in elderly women with reduced cortical bone thickness of the distal tibia should raise awareness for an osteoporosis assessment to prevent future osteoporotic fractures.

## Figures and Tables

**Table 1:** Overview of included trimalleolar ankle fractures. COPD = chronic obstructive pulmonary disease. SD = standard deviation.

Demographics		
	Age, year, mean, (SD)	56.4 ± 18.2
	Sex	139 female (72%) 54 male (28%)
Comorbidities		
	Nicotine abuse	36 (19%)
	Alcohol abuse	25 (13%)
	Diabetes	36 (19%)
	Malignancies	18 (9%)
	Cardiovascular disease	74 (38%)
	COPD/Asthma	13 (7%)
	Osteopenia/Osteoporosis	8 (4%)
Preinjury mobility		
	Unrestricted	168 (87%)
	Limited in sports	7 (4%)
	No longer walking distances possible	8 (4%)
	Mobile in the home	6 (3%)
	Bedridden	3 (1.5%)
	Unknown	1 (0.5%)
Need of aids		
	None	182 (94%)
	One walking cane	0 (0%)
	Two walking canes	1 (0.5%)
	Walker	4 (2%)
	Wheelchair	1 (0.5%)
	Unknown	5 (3%)

**Table 2:** Mechanism and type of injury.

Mechanism of Injury		
	Ankle distortion while walking	150 (78%)
	Ankle distortion during sport	20 (10.5%)
	Bicycle accident	16 (8%)
	Motorbike accident	3 (1.5%)
	Fall > 3 m	2 (1%)
	Road accident as a pedestrian	1 (0.5%)
	Road traffic accident	1 (0.5%)
**Type of injury**		
	Isolated injury	169 (88%)
	Polytrauma	3 (1.5%)
	Accompanying foot injuries	12 (6%)
	Ankle dislocation	143 (74%)
	Open fracture	9 (5%)
	Traumatic nerve injury	1 (0.5%)
	Traumatic vascular injury	0 (0%)

**Table 3:** Patients with secondary surgery after definitive fracture fixation.

Indication	Number of Patients (*n*)
Wound complication	2
Surgical site infection	2
Loss of reduction/secondary dislocation	1
Implant malposition	5
Syndesmotic screw removal	44
Fracture consolidation	38

**Table 4:** Multivariable regression analysis with CBTTC as the dependent endpoint.

Independent Variable	Regression Coefficient	*p*-Value	95% CI
Sex: female	−0.508	<0.001	[−0.739; −0.278]
Age	−0.009	0.002	[−0.149; −0.003]
BMI	0.022	0.012	[0.005; 0.039]

## Data Availability

The datasets generated and analysed during the current study are not publicly available due to them containing information that could compromise research participant privacy/consent but are available from the corresponding author on reasonable request.

## Supplementary Materials

The following supporting information can be downloaded at https://www.mdpi.com/article/10.3390/jcm12113666/s1. Table S1: multivariable regression analysis with ROI_1_ as the dependent endpoint; Table S2: multivariable regression analysis with ROI_2_ as the dependent endpoint.
