# Supplementary material for: Use of Distal Tibial Cortical Bone Thickness and FRAX Score for Further Treatment Planning in Patients with Trimalleolar Ankle Fractures
Source: J Clin Med. 2023 May 25;12(11):3666. doi: 10.3390/jcm12113666 (PMC10253613; doi:10.3390/jcm12113666)
Supplement: Supplementary file 1 [file jcm-12-03666-s001.zip › jcm-2398519-supplementary.pdf]

Supplements

Table S1: Multivariable regression analysis with ROI_1 as the dependent endpoint.

Independent variable	Regression coefficient	p-value	95%CI
Age	-2.325	<0.001	[-3.083; -1.565]
Sex: female	-18.02	0.241	[-48.248; 12.217]
BMI	1.089	0.263	[-0.826; 3.005]
No alcohol abuse	22.827	0.228	[-14.40; 60.055]

Table S2: Multivariable regression analysis with ROI_2 as the dependent endpoint.

Independent variable	Regression coefficient	p-value	95%CI
Age	-0.821	0.002	[-1.331; -0.311]
Sex: female	-28.423	0.008	[-49.320; -7.524]
No alcohol abuse	22.471	0.096	[-4.014; 48.957]
